# Electro-responsive actuators based on graphene

**DOI:** 10.1016/j.xinn.2021.100168

**Published:** 2021-09-24

**Authors:** Yong-Lai Zhang, Ji-Chao Li, Hao Zhou, Yu-Qing Liu, Dong-Dong Han, Hong-Bo Sun

**Affiliations:** 1 State Key Laboratory of Integrated Optoelectronics, College of Electronic Science and Engineering, Jilin University, 2699 Qianjin Street, Changchun 130012, China; 2 State Key Laboratory of Precision Measurement Technology and Instruments, Department of Precision Instrument, Tsinghua University, Haidian District, Beijing 100084, China

**Keywords:** Electro-responsive actuators, Graphene, Electrostatic actuation, Electrothermal actuation, Ionic actuation

## Abstract

Electro-responsive actuators (ERAs) hold great promise for cutting-edge applications in e-skins, soft robots, unmanned flight, and *in vivo* surgery devices due to the advantages of fast response, precise control, programmable deformation, and the ease of integration with control circuits. Recently, considering the excellent physical/chemical/mechanical properties (e.g., high carrier mobility, strong mechanical strength, outstanding thermal conductivity, high specific surface area, flexibility, and transparency), graphene and its derivatives have emerged as an appealing material in developing ERAs. In this review, we have summarized the recent advances in graphene-based ERAs. Typical the working mechanisms of graphene ERAs have been introduced. Design principles and working performance of three typical types of graphene ERAs (e.g., electrostatic actuators, electrothermal actuators, and ionic actuators) have been comprehensively summarized. Besides, emerging applications of graphene ERAs, including artificial muscles, bionic robots, human-soft actuators interaction, and other smart devices, have been reviewed. At last, the current challenges and future perspectives of graphene ERAs are discussed.

## Public summary

• Actuators enable controllable shape morphing under external stimulations
• Design principles and working performance of graphene ERAs have been summarized
• Emerging applications of graphene ERAs have been reviewed

## Graphical abstract

**Figure:**
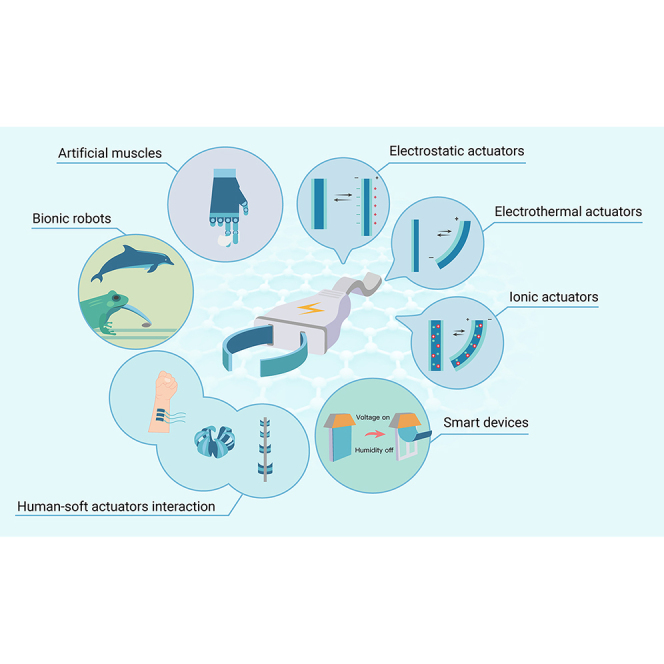

## Introduction

Stimuli-responsive actuators are devices that enable controllable shape morphing under the stimulation of external signals (e.g., light, temperature, pH, moisture, electric, and magnetic fields).^1^, ^2^, ^3^, ^4^, ^5^ As a core component for future smart devices, actuators are promising for cutting-edge applications in microfluidics,^6^, ^7^, ^8^, ^9^ robotics,^10^, ^11^, ^12^, ^13^, ^14^ aerospace,^15^, ^16^, ^17^, and biomedical science.^18^, ^19^, ^20^, ^21^, ^22^ Among various actuation methods, electro-responsive actuators (ERAs) have revealed distinct advantages over the others, including fast response, precise control, programmable deformation, and the ease of integration with control circuit or micro-electro-mechanical-systems (MEMS).^23^, ^24^, ^25^, ^26^, ^27^, ^28^, ^29^ Consequently, ERAs hold great promise for developing e-skins, soft robots, unmanned flight, and *in vivo* surgery devices.^30^, ^31^, ^32^, ^33^ According to different working mechanisms, ERAs can be classified into four kinds, including (1) electrostatic actuation, such as dielectric elastomer actuators, piezoelectric actuators, based on charge interactions^34^, ^35^, ^36^; (2) electrothermal actuation that makes use of *Joule* heating effect induced asymmetric thermal expansion between bi-/multilayer structures^37^, ^38^, ^39^; (3) ionic actuation that works through anions/cations induced asymmetric volume change of electrode layers^40^, ^41^, ^42^; and (4) electro-hydraulic actuation that integrated power electronics and motor drive.^43^, ^44^, ^45^ Generally, the structure of ERAs is simple, consisting of electrodes, electro-responsive materials, and coupled deformable materials/substrates. Recent advances in material science have pushed the rapid progress of ERAs forward, various functional materials, for instance, shape memory polymers,^42^, ^43^, ^44^ dielectric elastomers,^45^, ^46^, ^47^ piezoelectric materials,^46^, ^47^, ^48^ and carbon materials (e.g., nanotube and graphene)^49^ have been successfully employed to develop ERAs with high speed, high sensitivity, and wide-range response.

As a two-dimensional (2D) carbon material, graphene exhibits several unique properties,^50^, ^51^, ^52^ for instance, high carrier mobility (∼1.5 × 10^4^ cm^2^ V^−1^s^−1^),^53^, ^54^, ^55^ strong mechanical strength (Young's modulus, ∼1.0 TPa; tensile strength, ∼130 GPa),^56^^,^^57^ outstanding thermal conductivity (∼5.0 × 10^3^ W mK^−1^),^58^ high specific surface area,^59^^,^^60^ flexibility,^61^, ^62^, ^63^ and transparency.^64^^,^^65^ Consequently, graphene and related materials have emerged as appealing candidates in developing different kinds of electronic devices,^66^, ^68^, ^67^ including generators,^69^, ^70^, ^71^ supercapacitors,^72^, ^73^, ^74^, ^75^, ^76^ photodetectors,^77^, ^78^, ^79^ sensors,^80^, ^81^, ^82^ and actuators.^83^, ^84^, ^85^, ^86^, ^87^ Especially, after more than 15 years of intense research, graphene can be readily prepared by various approaches, such as chemical vapor deposition (CVD), reduction of graphene oxide (GO), and laser-induced graphene (LIG).^88^, ^89^, ^90^, ^91^, ^92^ Nowadays, due to the advances in preparation methods, graphene and its derivatives have been successfully employed in various stimuli-responsive actuators, revealing the great potential for adaptive optics, bionic robots, motion perception, and soft MEMS.^93^, ^94^, ^95^ In particular, considering the excellent electrical-/optical-/thermal-properties, significant progress has been made in graphene-based ERAs recently.

In this review, we have summarized the recent advances in graphene-based ERAs. The typical working mechanisms of ERAs that are made of graphene are introduced, in which the role of graphene in such ERAs has been highlighted. Also, the design principles (e.g., electrostatic actuators, electrothermal actuators, and ionic actuators) and unique properties of graphene-based ERAs are comprehensively reviewed. After that, the emerging applications of graphene-based ERAs, including artificial muscles, bionic robots, human-soft actuators interaction, and other smart devices, are comprehensively summarized. Last, the current challenges and future perspectives of graphene-based ERAs are discussed.

## Mechanisms

**Figure 1:**
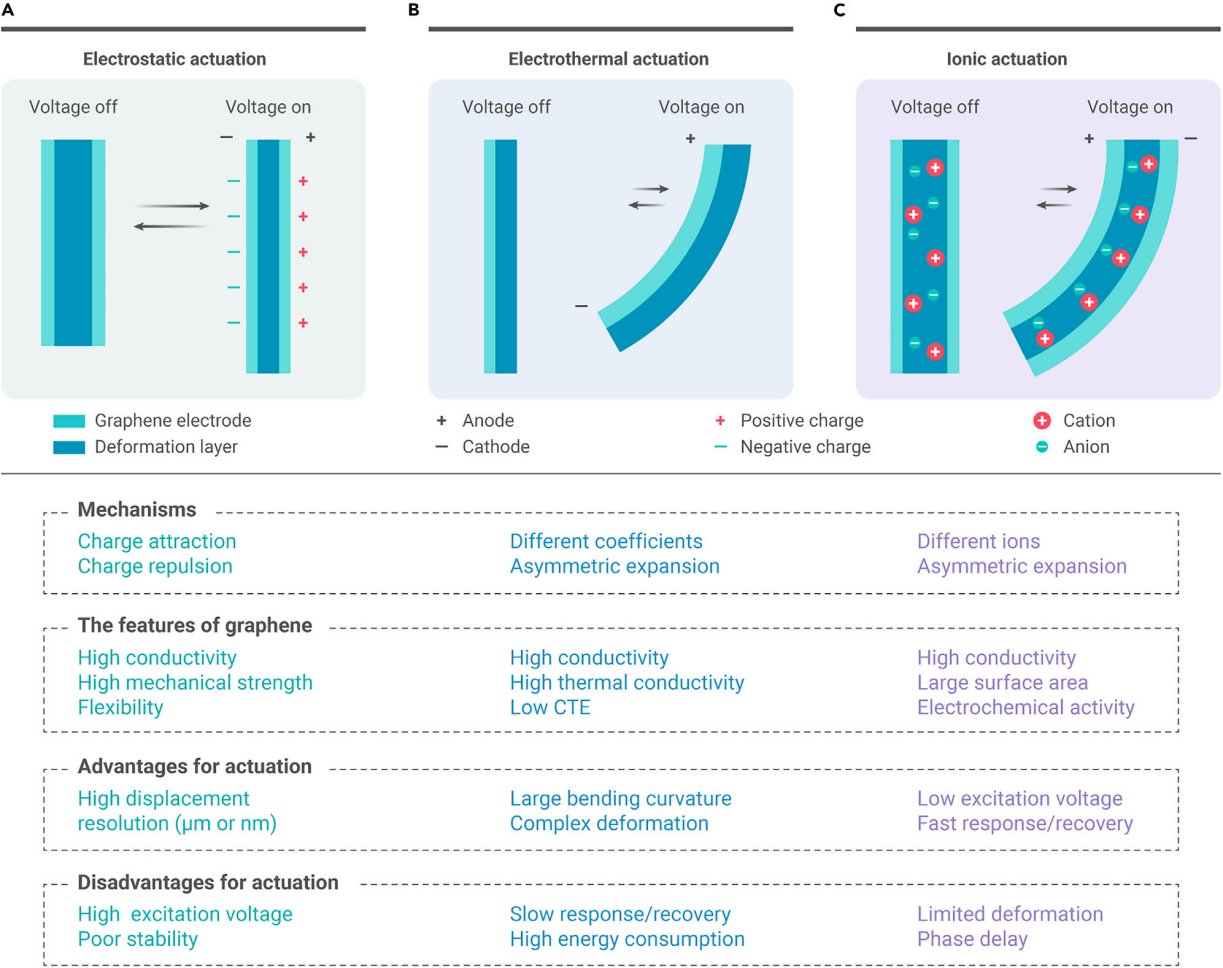
Schematic illustrating the mechanisms, features of graphene, advantages, and disadvantages for ERAs based on graphene (A) Electrostatic actuation. (B) Electrothermal actuation. (C) Ionic actuation.

ERAs can be designed and fabricated based on different working mechanisms. In this section, typical actuation strategies for graphene-based ERAs are introduced (Figure 1), including electrostatic actuation, electrothermal actuation, and ionic actuation. The unique advantages and disadvantages of such actuators and the role of graphene are discussed.

### Electrostatic actuation

Electrostatic actuation deforms by attraction or repulsion between charges (Figure 1A). For electrostatic actuation, graphene can serve as the electrode due to its excellent conductivity, stability, and mechanical strength. Electrostatic force can be generated when another electrode with the same or opposite charge approaches the graphene electrode. Generally, the electrostatic force is related to the applied voltage, the distance between two electrodes, and two electrodes' geometries. This kind of electrostatic actuation shows nanoscale high-accuracy displacements under dozens of voltages.^96^^,^^97^ To improve response/recovery time and actuation displacement, dielectric elastomer actuators, and piezoelectric actuators have been widely developed. For example, the dielectric elastomer actuator is sandwiched with two flexible electrodes. The charges separate on the elastomers and electrode interface under actuation. Therefore, electrostatic force formed on the elastomer layer's thickness direction leads to thinning thickness and actuation. Graphene can be added into the dielectric elastomer to improve the dielectric constant, leading to quick electrostatic response and large bending force.

### Electrothermal actuation

Typically, electrothermal actuation is based on bi-/multilayer structures (Figure 1B), in which an electricity-to-thermal layer connects to the external power source. Different layers own different coefficients of thermal expansion (CTE). Then the electricity-to-thermal layer converts electricity to joule heat and raises the localized temperature of the entire structure. Electrothermal actuators bend to the low-CTE layer. Notably, the CTE of graphene is lower than that of ordinary metal and polymer materials. For graphene-based electrothermal actuators, graphene has two typical functions: (1) working as the conductive material in low-CTE layer (inert layer) to reduce system power consumption (high conductivity and low CTE); and (2) serving as the dopant in the high-CTE layer to improve the response/recovery time (high thermal conductivity). Graphene-based electrothermal actuators have edges on large bending curvatures because of higher voltage and adequate materials expansion.

Furthermore, electrothermal actuators can achieve complex three-dimensional (3D) deformations by rational structure design. Nevertheless, graphene-based electrothermal actuators require high applied voltage (dozens of voltages) to achieve large bending curvature and suffer from low response/recovery speed because the thermal expansion/contraction rate of materials (electrothermal actuation) is relatively low. Besides, the mechanical stability of electrothermal actuators needs to be improved because frequent bending/unbending may induce interlayer shedding.

### Ionic actuation

Ionic actuators contain two electrodes and an electrolyte layer (Figure 1C). Under actuation, the cations migrate and insert into the cathode along with the thickness of ionic actuators. Meanwhile, the anions migrate and insert into the anode. The migration and insertion of ions induce the expansion of both electrodes. Because of different radii of cations and anions, asymmetric expansion occurs between two electrode layers and leads to the bending of whole ionic actuators to the anode side. Therefore, the electrodes of ionic actuators require high conductivity and good contact between electrode materials and electrolyte materials. Traditionally, the electrode materials of ionic actuators are noble metal materials, such as silver and platinum, but the noble metal electrode may suffer from high cost, poor flexibility, and environmental adaptability. For example, the device's lifetime may seriously decrease by using noble metal electrodes because the noble metal film can break after multiple bending/unbending and under a humidity environment. Consequently, researchers are always seeking new robust and conductive materials to develop high-performance ionic actuators at a low cost. Graphene features high conductivity, flexibility, large surface area, extraordinary mechanical strength, and chemical/physical stabilities, revealing great potential for practical applications as electrodes for high-performance ionic actuators. More importantly, as compared with other electrodes, graphene demonstrated more significant volume expansion (∼700%) under ion insertion, leading to larger deformation.^98^ Typically, due to these excellent properties, graphene-based ionic actuators show low actuation voltage (<1 V), fast response (<1 s), and excellent stability; however, in the process of development of graphene-based ionic actuators, disadvantages also exist, like noble metal electrode materials, such as phase-delay and limited deformation (only bending). It can be believed that graphene has immeasurable development potential in the field of ionic actuators.

### Design principle and performance of ERAs

#### Electrostatic actuators

**Figure 2:**
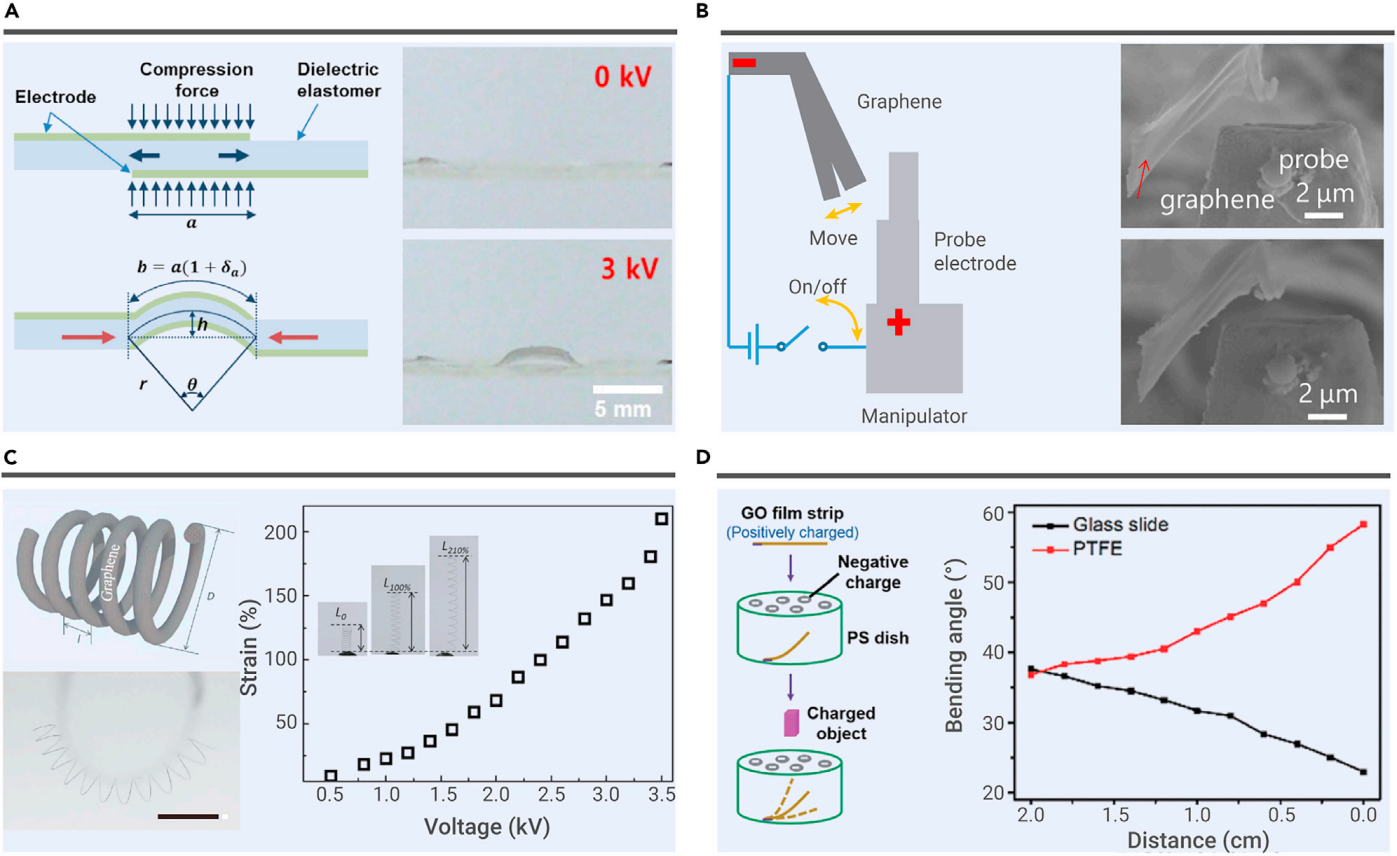
Electrostatic actuation (A) Schematic illustrating the behaviors of the dielectric elastomer actuators (V = 0, 3 kV). Reproduced with permission from Kim et al.^99^ Copyright 2013, IOP Publishing Ltd. (B) Electrostatic actuation between graphene and probe electrode. Reproduced with permission from Fujiwara et al.^103^Copyright 2016, IEEE. (C) Functional graphene springs for electrostatic actuation. Reproduced with permission from Cheng et al.^105^ Copyright 2014, The Royal Society of Chemistry. (D) Graphene oxide film-based electrostatic actuators. Reproduced with permission from He et al.^106^ Copyright 2017, The Royal Society of Chemistry.

Electrostatic actuators show displacement by the attraction or repulsion between charged objects. For example, Kim et al. developed graphene-optically clear adhesive-graphene (G-OCA-G) sandwiched dielectric elastomer actuators by roll-to-roll methods.^99^ The electrostatic force creates at the OCA film and graphene electrode interfaces and squeezes the OCA in thickness under actuation (Figure 2A). As a result, the G-OCA-G dielectric elastomer actuator shows a large displacement of ∼1050 μm by applying the voltage (3 kV, 0.5 Hz) because graphene provides high electrical conductivity and considerable elasticity. Besides, graphene and OCA film show high transparency. Therefore, this work offers a novel way to develop electrostatic actuators with high transparency (∼84.5%). Similarly, Bae et al. replaced the dielectric elastomer with poly(vinylidene fluoride-co-trifluoroethylene) (P[VDF-TrFE]) and created a piezoelectric actuator based on G-P(VDF-TrFE)-G device structures.^100^ Instead of acting as the electrode materials in dielectric elastomer actuators, graphene also plays a vital role in improving the dielectric elastomer's dielectric constant. As a successful example, Zhang et al. fabricated graphene-polydimethylsiloxane/polydimethylsiloxane (G-PDMS/PDMS) composite film for dielectric elastomer actuators.^101^ The displacement of the G-PDMS^3^/PDMS film is 1.12 mm at 63.4 MV/m.

In addition to the dielectric elastomer actuators, Fujiwara et al. demonstrated electrostatic actuation by applying the voltage between a probe electrode and the multi-graphene.^102^^,^^103^^,^^104^The distance of probe and multi-graphene changed 0.67 μm under a voltage of 110 V because of the attraction between the graphene and electrode (Figure 2B). To achieve higher displacements, Cheng et al. creatively demonstrated reduced GO (RGO) springs for electrostatic actuation (Figure 2C).^105^ The RGO springs are achieved by wrapping wet RGO fibers around cylindrical objects and annealing under high temperatures (500–800°C). The diameters and loop distance of RGO springs can be controlled by varying internal cylinders and winding densities. The RGO springs process high-strain capability and low elasticity coefficient ([4.6–6.8] ×10^−4^ N m^−1^). Besides, the RGO springs show a linear relationship between the strain (within 300%) and applied force (∼0.04 mN). Therefore, due to the repulsion between the charged loops, RGO springs process approximate linear length changes when the applied voltage is raised from 0 to 3.5 kV. Remarkably, the RGO springs show excellent elongate rates (ca. 210% per s) under an electrostatic field (3.5 kV), showing potentials in developing electrostatic switches.

To develop contactless actuators, He et al. fabricated contactless GO strip-based electrostatic actuators by attaching a positively charged GO strip on a negatively charged polystyrene (PS) Petri dish (Figure 2D).^106^ The positively charged GO strip is attracted by the negatively charged dish leading to a certain bending angle. Due to the extraordinarily lightweight and flexible characters of GO, the GO strip is sensitive to external charged materials and can be actuated by weak attraction or repulsion force (less than 1 nC). Consequently, the bending angle increased when negatively charged materials, such as polytetrafluoroethylene, PS, approach the GO strip. Simultaneously, the bending angle decreased when positively charged materials, such as glass, weighing papers, or steel, approach the GO strip. Surprisingly, the GO strip shows a fast response to human fingers because of the electrostatic forces. The GO strip bends to 39.5° in 0.56 s when a human finger moves closer. The GO strip recovers to the initial bending angle in 0.75 s without actuation. This fast actuation response to human fingers shows potentials in developing contactless actuators.

#### Electrothermal actuators

As we know, electrothermal actuators are typically bi-/multilayer structures. The whole structure bends because of the electrothermal stress mismatches at the interface of different layers. For example, Liang et al. successfully fabricated RGO/polydiacetylene (PDA) bilayered electrothermal actuators by the thermal-/electric-induced expansion of PDA.^107^ The RGO can transfer electric energy into thermal energy to heat the bilayered electrothermal actuators. The CTE of PDA (1 × 10^−4^ K^−1^) is larger than RGO (30 × 10^−6^ K^−1^).^108^^,^^109^ Besides, electric-induced expansion of PDA crystal also plays a vital role in actuation. Consequently, RGO/PDA electrothermal actuators achieved large curvature (0.37 cm^−1^) by applying a low current density (0.74 A/mm^2^). It is worth noting that the RGO/PDA actuators show ultrafast switch behaviors even up to 200 Hz under an alternating current, which may contribute to fabricating artificial muscles and robots.

**Figure 3:**
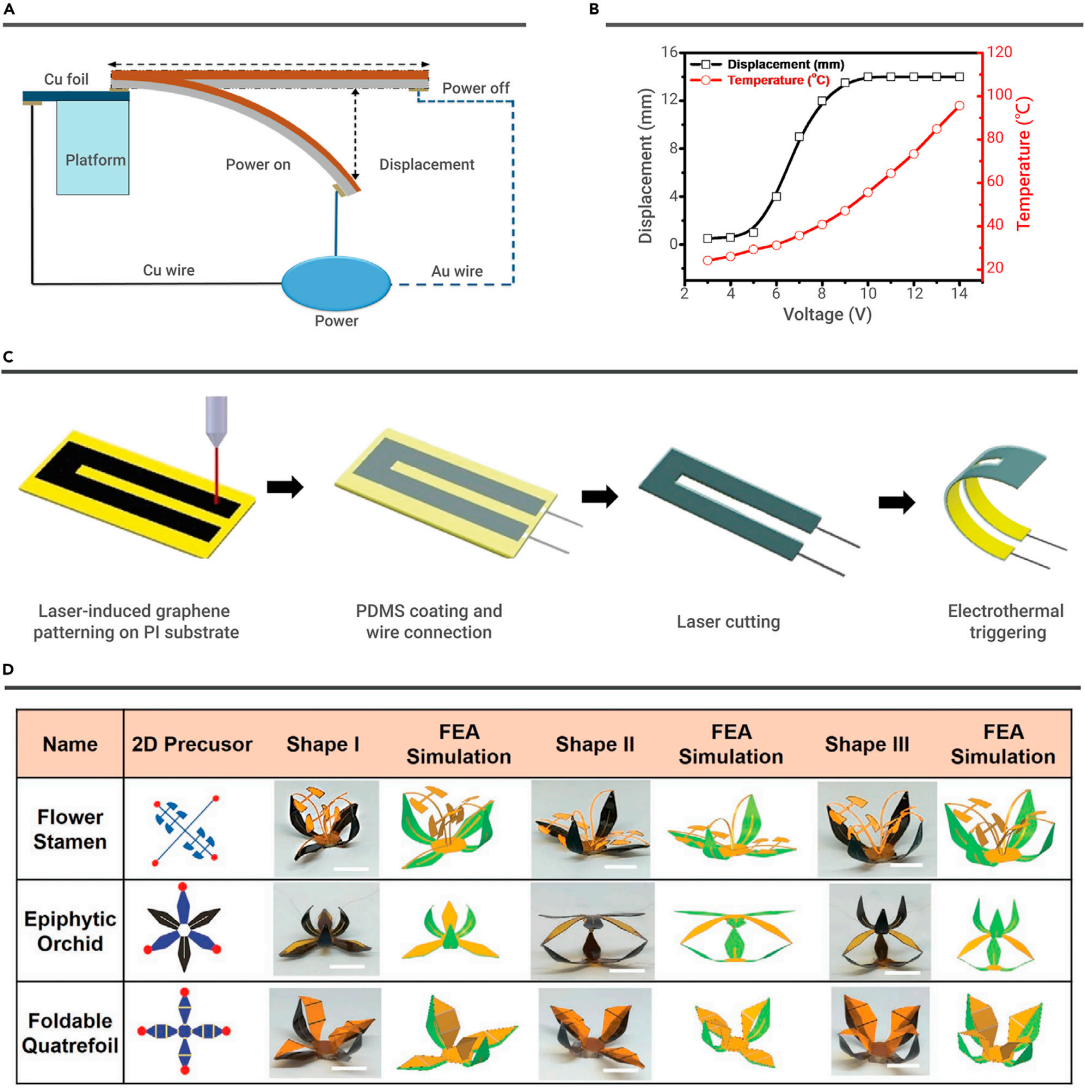
Electrothermal actuation (A and B) (A) The experimental setup for measuring electrothermal actuation displacement and (B) the displacement and temperature changes of RGO/PVDF bimorph actuators under different applied voltages. Reproduced with permission from Xiao et al.^110^ under the terms of the CC-BY Creative Commons Attribution 4.0 International License. Copyright 2016, The Authors, Published by Wiley-VCH. (C and D) (C) Fabrication of LIG-based electrothermal actuators and (D) 3D structures assembled by using LIG-based electrothermal actuators. Reproduced with permission from Ling et al.^39^ Copyright 2020, WILEY-VCH.

To improve the actuation performance, Xiao et al. chose large CTE materials (polyvinylidene fluoride [PVDF]) as the electric-induced expansion layer and fabricated RGO/PVDF electrothermal actuators (Figure 3A).^110^ The tip displacement of RGO/PVDF electrothermal actuators can reach 14 mm in 0.262 s by applying the voltage of 13 V (Figure 3B) because PVDF possesses large CTE (130 × 10^−6^ K^−1^), excellent electrostrictive performance, and piezoelectric effect performance.^111^, ^112^, ^113^, ^114^ Besides, various electrothermal actuators with high-CTE layer have been developed to bilayered electrothermal actuators, such as graphene/epoxy,^115^ RGO/GO,^116^ RGO/PDMS,^117^, ^118^, ^119^ RGO/polyimide (RGO/PI),^120^ and iodine-doped RGO/RGO.^121^

In recent years, laser processing has become a versatile technology to prepare graphene, including laser-reduced GO (LRGO) and LIG.^50^ Meanwhile, laser technologies are capable of high-efficiency, mask-free patterning, and hierarchical structuring of graphene, which is of benefit to the design and fabrication of electrothermal actuators. As typical examples, RGO- or LIG-based electrothermal actuators, including LRGO/PI,^122^ Ag-LRGO/PI,^123^ LRGO/polyethylene (LRGO/PE),^124^ PI/LIG/PVDF,^125^ GO/RGO/tape,^126^ and PI/LIG/PDMS^39^ structures have been successfully developed, demonstrating sophisticated deformation ability. The hierarchical structure of graphene plays an important role for the performances of ERAs. For instance, by tailoring graphene into hierarchical structures, the resultant electrothermal actuator exhibits a right- or left-handed helix deformation.^124^^,^^125^ Typically, Ling et al. developed a tri-layered electrothermal actuator based on the PI/LIG/PDMS structure (Figure 3C).^39^ The PI/LIG/PDMS electrothermal actuators are prepared by the following procedures: (1) laser patterning LIG on PI membranes, (2) spin-coating and curing PDMS on the PI membrane, and (3) cutting into different shapes. The LIG produces Joule heating and introduces temperature differences between the PI and PDMS layer by applied electric field. Therefore, the PI/LIG/PDMS electrothermal actuator bends to the PI sides because the PI layer processes lower CTEs than the PDMS layer. The maximum bending curvature is 3.3 cm^−1^ (V = 30 V). Instead of producing joule heating, the LIG has porous structures after the laser treatment, contributing to the PDMS penetration into porous structures and enhancing the interfacial adhesion between the LIG and PDMS layers. As a result, the PI/LIG/PDMS electrothermal actuators show no significant bending/unbending performance degradation over 1000 times. More importantly, inspired by origami creases and kirigami cuts, this method has realized 2D to 3D shape deformations through global folding and local bending by laser thinning (thickness control) and laser cutting (cutting patterns control) technologies. Impressively, more than 20 predetermined 3D architectures have been designed and fabricated (Figure 3D). This work provided a feasible way to realize 2D to 3D complex shape deformations.

#### Ionic actuators

**Figure 4:**
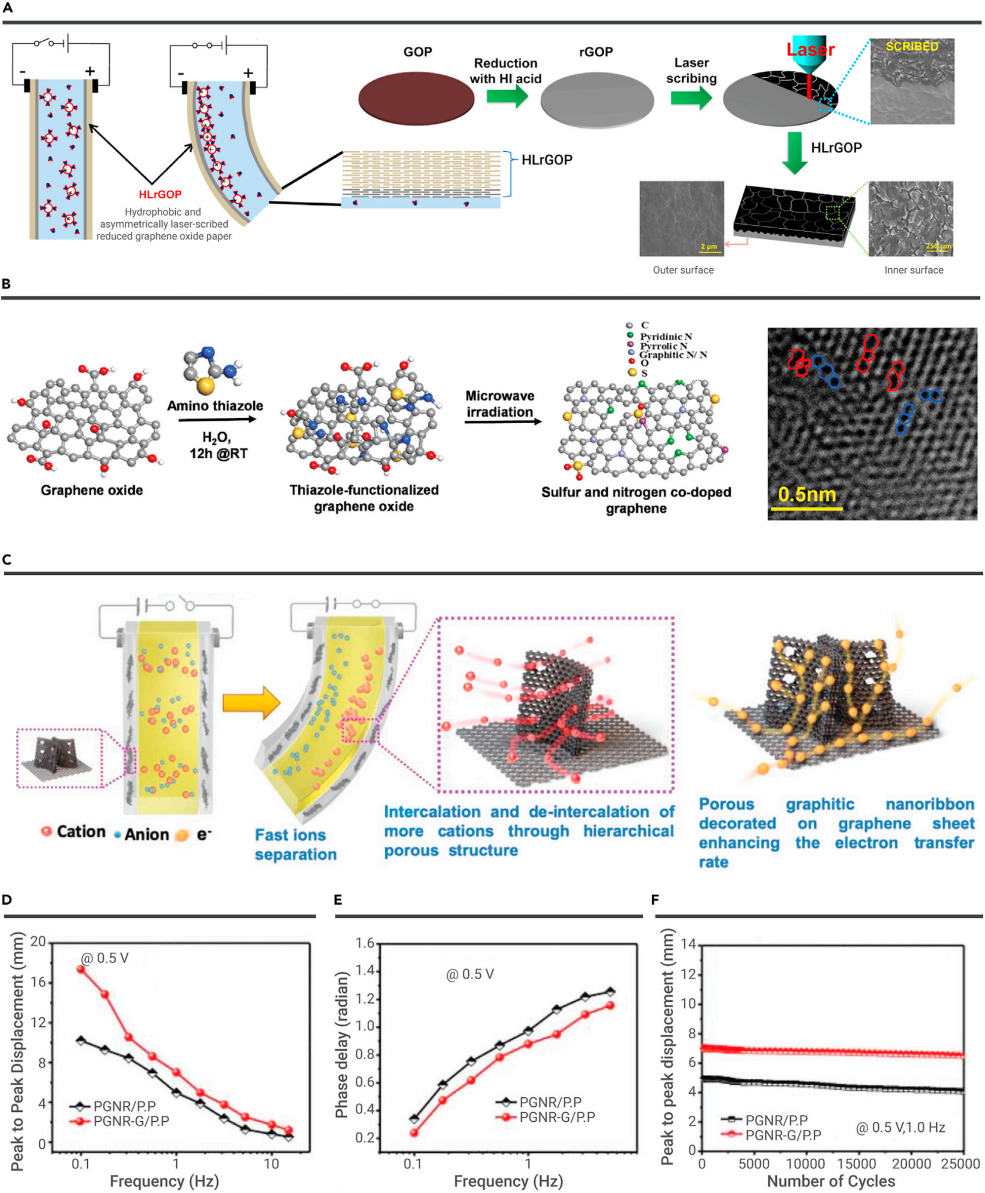
Ionic actuation (A) Schematic illustrating the mechanisms and the preparation process of LRGO electrodes for ionic actuators. Reproduced with permission from Kim et al.^127^ Copyright 2014, American Chemical Society. (B) The concept of sulfur and nitrogen co-doped RGO electrodes for ionic actuators and the corresponding HRTEM image of sulfur and nitrogen co-doped RGO. Reproduced with permission from Kotal et al.^129^ Copyright 2016, WILEY-VCH. (C–F) (C) Schematic illustrating the actuation mechanism of ionic actuator with 3D hetero-nanostructured RGO electrode. The corresponding (D) bending responses, (E) phase-delay responses, and (F) cyclic stabilities. Reproduced with permission from Kotal et al.^140^ Copyright 2020, WILEY-VCH.

In this section, we summarize the fabrication methods and unique characters of graphene-based ionic actuators. Typically, Kim et al. fabricated laser-scribed-RGO (LS-RGO) paper electrodes asymmetrically for highly durable ionic polymer-graphene composite actuators (Figure 4A).^127^ For LS-RGO paper preparation, GO papers are first reduced by HI acid solution to produce highly conductive and smooth HI-RGO (sheet resistance, ∼6.35 Ω/sq). The outer smooth hydrophobic HI-RGO surface can prevent the leakage of the electrolyte during actuation. Then, the HI-RGO papers are laser-scribed on the inner surface, and the outer remains smooth surfaced. After the laser-scribing process, the LS-RGO surface is a porous structure, contributing good adhesion with the ionic polymer (i.e., Nafion). As a result, the tip displacement of graphene-based ionic actuators is up to 4.26 mm, and the maximum bending curvature is 8.75 m^−1^ under 5 V @ 0.01 Hz. The normalized tip displacement maintains above 90% after 6 h, whereas the tip displacements of conventional ionic polymer-metal composite actuators drop rapidly below 50% after 6 h. This work fully indicates the advantages of the graphene electrode layer compared with the traditional noble metal electrode layer. In addition, Tabassian et al. proposed a self-sensing ionic actuator, which senses bending deformation under actuation.^128^ The self-sensing ionic actuator is fabricated by embedding a graphene mesh electrode inside the ionic polymer layer. Under actuation, the graphene mesh traces the migration of mobile ions inside the ionic polymer layer, which proves to be related to the bending deformation of the actuator.

To improve graphene-based ionic actuator performances, various strategies, including heteroatom doping, material compositing, and hierarchical structuring, have been developed to improve conductivity and provide active sites for electrochemical reactions. Kotal et al. fabricated sulfur/nitrogen co-doped RGO (SNG) by a microwave-associated method and developed SNG/polyethylenedioxythiophene: polystyrenesulfonate (SNG/PEDOT:PSS) electrodes to enhance electrochemical activities (Figure 4B).^129^ Compared with RGO, as the high-resolution transmission electron microscope (HRTEM) image shown, sulfur/nitrogen co-doping-induced structural defects include pentagon, heptagon, and even 9-membered rings in graphene honeycomblike lattice structure, which play an important role in facilitating electrolyte diffusion. Besides, the sulfur/nitrogen co-doping enhances the charge density and the binding interaction with ions. Therefore, the intrinsic resistance of the RGO decreases. The electron transfer rate is improved. As a result, the capacitance of SNG (284 F/g) is about 1.96 times the capacitance of RGO (145 F/g). Taking advantage of excellent electrochemical activity, the SNG/PEDOT:PSS-based ionic actuators show remarkable bending performance. The tip displacement of SNG/PEDOT:PSS-based ionic actuators is 4.5 mm under the excitation of ±1 V @ 0.1 Hz. More importantly, the bending performance remains 96% of initial strain over 18,000 cycles. Similarly, various graphene-based composite materials, such as RGO/Ag,^130^ RGO/Fe_3_O_4_,^131^ RGO/carbon nanotube (RGO/CNT),^132^,^133^^,^ nickel oxide nanowalls@RGO–multiwalled carbon nanotubes,^134^ graphene/PS,^135^ RGO/polyaniline,^136^ RGO/polypyrrole,^137^ and nSNRGO/pMoS_2_,^138^ have been achieved to act as electrodes for perfect actuation performance.

It is worth noting that Kotal et al. recently reported nitrogen-enriched 3D g-C_3_N_4_/nitrogen-doped graphene^139^ and 3D porous graphitic nanoribbon-graphene/PEDOT:PSS (PGNR-G/PEDOT:PSS)^140^ hetero-nano-architectured electrodes for ionic actuators. The 3D hetero-nanostructure enables unimpeded ion/electron channels and multidirectional electron transport (Figure 4C). As a result, 3D PGNR-G/PEDOT:PSS electrodes ionic actuators achieved a larger tip displacement (17.4 mm), a fast response time (700 ms), and excellent cycling stability under voltage of 0.5 V (Figures 4D–4F). Such 3D hetero-nano-architectured electrodes for ionic actuators open a new way to develop ultralow voltage-driven ionic actuators with excellent performance.

### Potential applications

#### Artificial muscles

**Figure 5:**
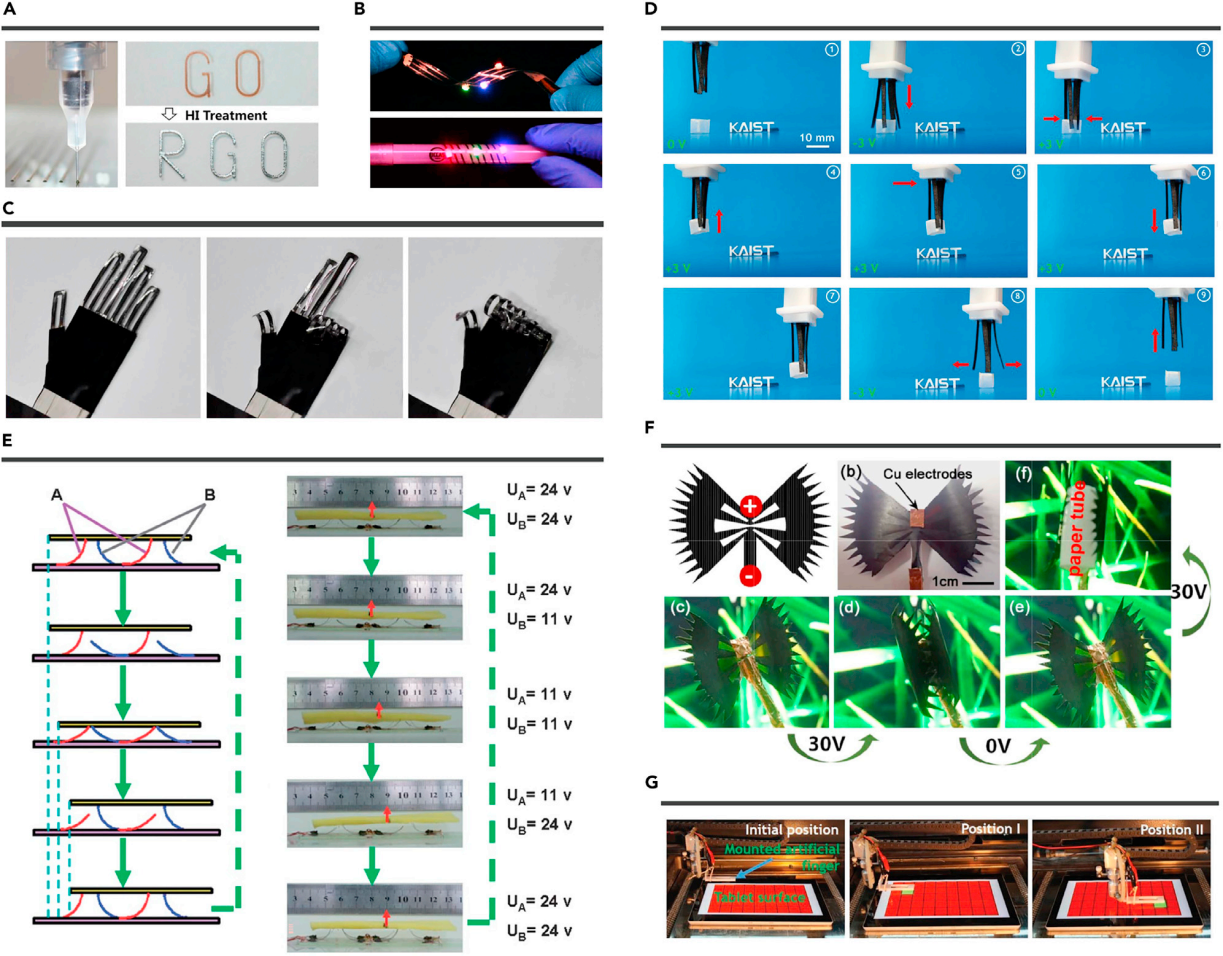
Artificial muscles (A) RGO patterning was fabricated by ink-printing and reduced technology. (B) Flexible RGO circuits. (C) Hand-shaped actuator. Reproduced with permission from Li et al.^118^ Copyright 2016, American Chemical Society. (D) Operation of a grapple robot. Reproduced with permission from Tabassian et al.^143^ Copyright 2018, WILEY-VCH. (E) Artificial cilia for moving objects. Reproduced with permission from Bi et al.^116^ Copyright 2013, The Royal Society of Chemistry. (F) Bionic flytrap robot. Reproduced with permission from Zhu et al.^124^ Copyright 2019, Optical Society of America. (G) Soft finger performing touching task. Reproduced with permission from Manzoo et al.^138^ Copyright 2019, WILEY-VCH.

Natural muscle can contract or expand to cause movement. In recent years, various excellent works have been developed to fabricate high-performance artificial muscles. As a pioneering work, Li et al. fabricated hand-shaped RGO/PDMS electrothermal actuators by combining direct ink writing techniques with chemical reduction.^118^ The GO patterns were first ink-printed on the PDMS surface (Figure 5A). Then, the GO pattern was reduced by HI to prepare RGO. RGO shows excellent conductivity (∼4.51 [±0.18] × 10^4^ S/m). Interestingly, the light-emitting diode chips integrated with RGO circuits keep bright illumination upon bending and twisting (Figure 5B). The RGO/PDMS electrothermal actuators (10-mm length) bends to 300° in 5 s (V = 13.5 V) and recovers to the initial position in 9 s. Taking advantage of patterning design, hand-shaped RGO/PDMS electrothermal actuators were fabricated. An independent power system controls each finger. Therefore, the hand-shaped RGO/PDMS electrothermal actuators show various programmable control gestures (Figure 5C). Notably, different complex deformation may be realized by the printing strategy and versatile design.

Significantly, the bending motions show abilities in kicking, hooking, and grasping.^117^^,^^120^^,^^123^^,^^125^^,^^142^,^141^^,^ For example, Tabassian et al. fabricated high-performance grapple robots consisting of four ionic actuator arms; the hybrid electrodes are composed of hollow tubular graphene mesh (GM) and N-doped crumpled RGO (NG).^143^ The GM shows a highly conductive network and low capacitance, suitable for the fast and uniform charge distribution. NG has low conductivity and high capacitance, which helps the charge storage. The two materials' synergic effect increased the maximum displacement by ∼620% (a square wave signal) and 380% (a sine wave signal), respectively. Additionally, the output force is ∼1.2 mN by applying a direct current (DC) voltage of 3 V and increases linearly with the input voltage. To fabricate a grapple robot (Figure 5D), four GM-NG ionic actuators are assembled around a cubic electrode. The grapple robot can lift a paper cube and carry the paper cube to another position. This work integrates the driving component with moving arms, leading to reduce the device size efficiently. Therefore, this grapple robot shows potential in developing surgical robot use that requires the grapple robot to transport small things in narrow spaces. Bi et al. fabricated artificial cilia that can be used to move objects 9 mm in one cycle (Figure 5E).^116^ Besides, inspired by the bionic flytrap, flytrap-shaped actuators were developed based on the RGO/PE electrothermal actuators^124^ and 3D PGNR-G/PEDOT:PSS ionic actuator^140^ for trapping and consuming insects under actuation (Figure 5F). Impressively, Fujiwara et al. fabricated nano-grippers by a focused ion beam process.^103^ Initially, multilayered graphene (thickness, 0.9 μm), prepared by mechanical exfoliation, was fixed on the sample stage. Then groove lines were prepared by the focused Ion beam (FIB) etching. After the FIB etching, the multilayered graphene tip shows opening and closing motions upon different electrostatic forces. The nano-grippers are actuated inside a field emission scanning electron microscope. This work indicates the promising applications in integrating graphene into nano electro mechanical system.

Instead of shape deformation, Manzoor et al. developed soft-touch fingers ionic actuators for touching smartphones and tablets.^138^ The touch fingers ionic actuators are prepared by the hybrid electrode (pMoS_2_-nSNRGO) with high conductivity and high capacitance. Accordingly, the pMoS_2_-nSNRGO–based ionic actuators show larger displacement (670% improvement) than RGO-based ionic actuators at 0.5 V and 1 Hz. The low actuation voltage may play an essential role in connecting actuators into control systems for practical applications. The soft-touch fingers can turn on/off the smartphone's flashlight. Besides, as shown in Figure 5G, the toughing function on tablets can be realized by mounting the soft-touch fingers on a moving stage. This work shows that ionic actuators can perform more effectively by connecting with control circuits and systems.

#### Bionic robots

The design of ERAs based on graphene can be used to design insect-like motions, such as flapping motions, moving movements, and even preying biomimetic frog tongues.

##### Flapping motions

**Figure 6:**
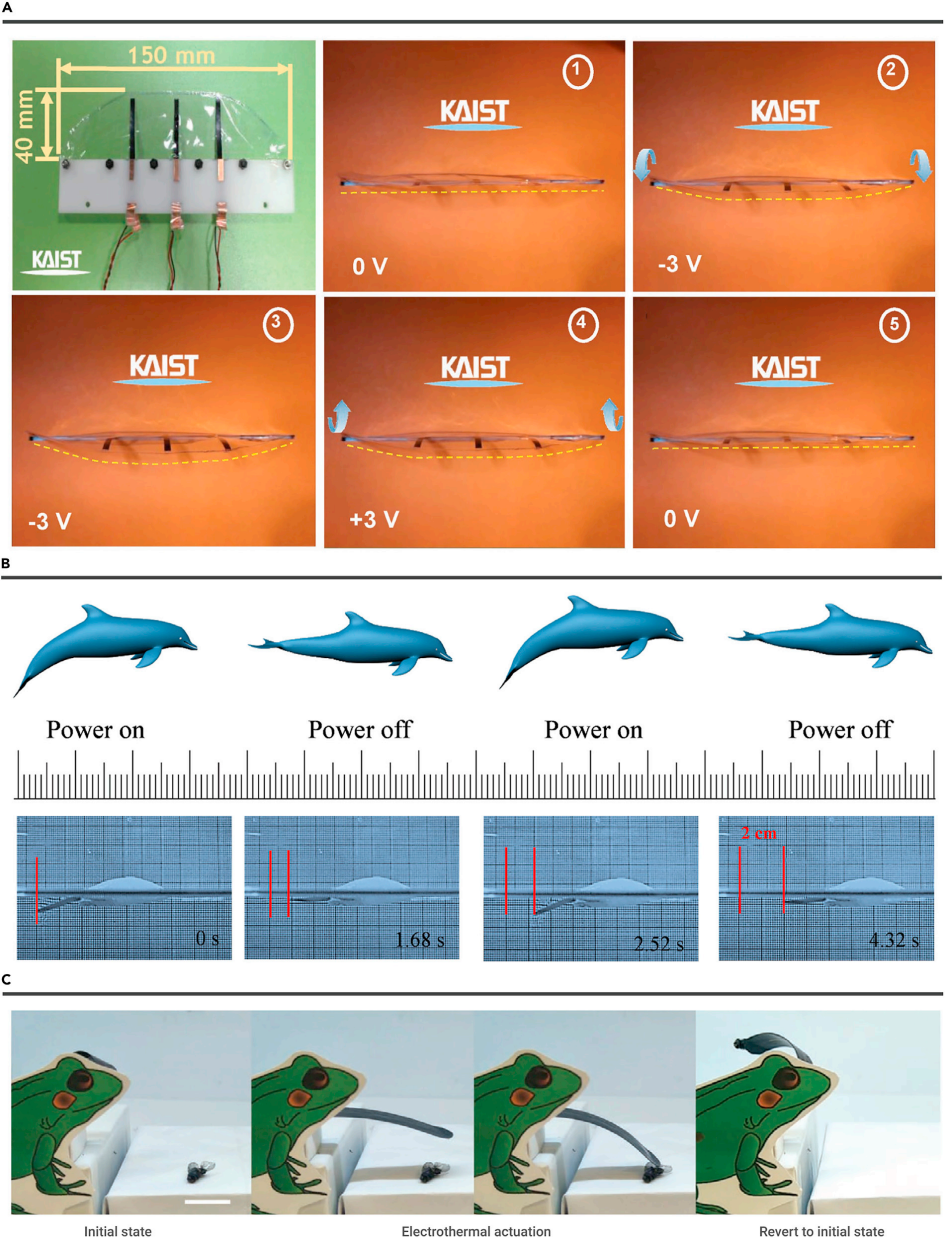
Insect-like motions (A) An artificial fish fin. Reproduced with permission from Kotal et al.^139^ Copyright 2018, WILEY-VCH. (B) The fish-like robot swimming. Reproduced with permission from Xiao et al.^110^ under the terms of the CC-BY Creative Commons Attribution 4.0 International License. Copyright 2016, The Authors, Published by Wiley-VCH. (C) A biomimetic frog tongue prey insect. Reproduced with permission from Ling et al.^39^ Copyright 2020, WILEY-VCH.

Zhu et al. developed graphene/SU-8-based micro-electrothermal actuators for transparent dragonfly wings.^115^ The graphene was patterned by photolithography and O_2_ plasma reactive ion etching. Then the graphene/SU-8-based micro-electrothermal actuator bends to the graphene side by applying the electric actuation. Additionally, the graphene/SU-8-based micro-electrothermal actuators show a displacement of 1 μm in 0.02 s by using a 1 V and recovery voltage to the initial position in ∼0.1 s. The efficient bonding (π-π interaction) between graphene and SU-8 helps avoid delamination up to 0.4% strain.^144^ Besides, graphene is flexible and low weight, which is suitable to develop wings. As a result, graphene/SU-8–based micro-electrothermal actuators are used to develop dragonfly wings with flapping motions. The flapping movements can be controlled by varying frequencies and durations of the applied voltage. Instead of developing wings, Kotal et al. demonstrated artificial fish fins by aligning three ionic actuators in a row (interactor distance, 30 mm) and attaching low-density polyethylene membranes on ionic actuators (Figure 6A).^139^ The ionic actuators (length, 18 mm; width, 3 mm) exhibit a large bending strain of 0.52% and high duration (93% retention) after 5 h of actuation. As a result, the artificial fish fin shows harmonic movements by applying harmonic input.

##### Moving forward motions

Xiao et al. demonstrated a fish-like robot consisting of a polystyrene body (30 mm × 8 mm) and an RGO/PVDF actuator (14 mm × 3 mm) based tail (Figure 6B).^110^ The RGO/PVDF actuator bends down under 13 V @ 0.4 Hz and offers propulsion. Besides, the RGO/PVDF actuator recovers the initial positions after power is turned off. As a result, the fish-like robot swims forward (v = 5.02 mm/s). The bending movement can be controlled by values and frequencies of applied alternating current (AC) and actuator size, showing great potentials in developing moving motions. As a pioneer work, Liang et al. developed a bidirectional moving robot based on RGO/PDA actuators with asymmetrical shapes.^107^ The bidirectional moving robot consists of a large part (∼18 mm^2^) and a small part (∼8 mm^2^). The small part produces a larger amplitude than the large part (±20 mA @ 50 Hz). Therefore, the bidirectional moving robot moves from right to left due to the different amplitude between the large and small parts. On the contrary, the large part produces a larger amplitude than the small part (±16 mA @ 50 Hz). As a result, the bidirectional moving robot moves from left to right.

##### Preying of biomimetic frog tongues

Unlike the works mentioned above in this section, Ling et al. recently fabricated a biomimetic frog tongue that is capable of capturing flies under 20 V actuation (Figure 6C).^39^ The bidirectional shape transformations are manufactured by inducing thermal stress during the fabrication process. Initially, the frog tongue rolls up (∼360°) toward the PDMS side without actuation. Then, the frog tongue unbends (∼300°) toward the PI to capture a fly under actuation. The PI/LIG/PDMS tri-layered structure recovers to the initial state without actuation.

#### Human-soft actuators interaction

**Figure 7:**
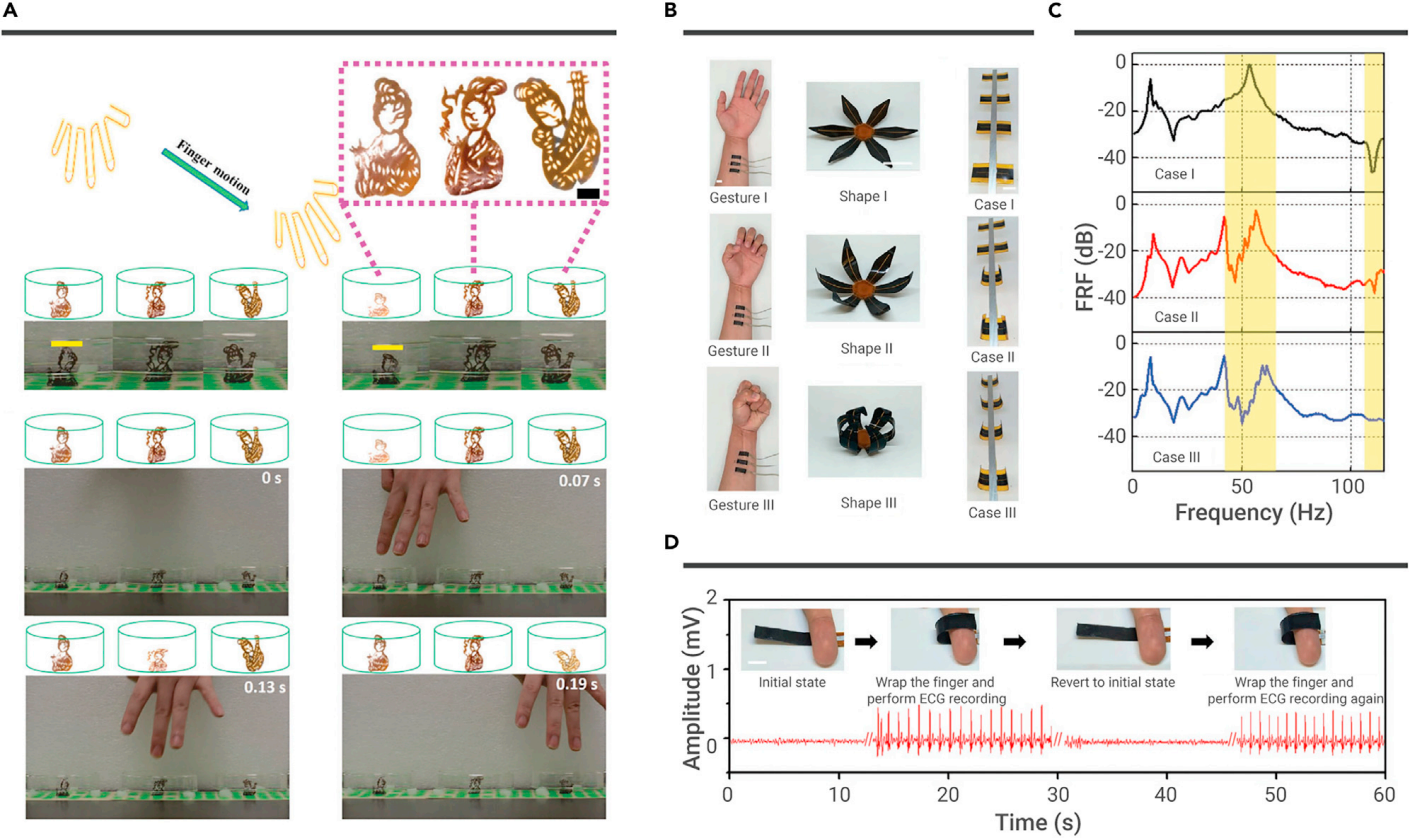
Human-soft actuators interaction (A) GO film-based dancers. Reproduced with permission from He et al.^106^ Copyright 2017, The Royal Society of Chemistry. (B and C) (B) Human gestures can be used to control flower-like structure and an elastic metamaterial for (C) tunable bandgap behaviors. (D) A LIG-based actuator for electrocardiogram (ECG) measurement. Reproduced with permission from Ling et al.^39^ Copyright 2020, WILEY-VCH.

He et al. demonstrated GO film-based radar and dancers, showing great potentials in developing human motion monitors.^106^ Due to the electrostatic force between GO films and human fingers, the film's bending degree increases when a finger approaches the film. Taking the GO film-based radar as an example, a radar was fabricated by arraying 3 × 3 GO strips with a grid size of 1.9 cm. The GO strip closest to the finger has maximum bending degree changes when it is above the radar. As the finger moves, the GO strip with the maximum bending angle changes accordingly. Therefore, the finger's position information can be inferred from the GO strip's maximum bending angle changes. Meanwhile, GO film-based dancers with traditional Chinese human images are also developed by Chinese paper cuts (Figure 7A). Initially, the GO film-based dancers (width × height, ∼1.6 cm × 2.8 cm) remain in a standing state. The bend motion occurs when the finger is close to the GO film-based dancer. The GO film-based dancer immediately recovers to the initial unbent state when the finger moves away from the GO film-based dancer. It is worth noting that GO film-based dancers with traditional Chinese human images produce the dancing process when different fingers repeatedly approach and leave from GO film-based dancers.

Instead of the electrostatic force directly inducing shape deformation, Ling et al. achieved human gestures for controlling actuators' 3D shapes via real-time monitoring of human electromyogram (EMG) signals (Figure 7B).^39^ LIG-based electrophysiological sensors are attached to the forearm of a human for recording the EMG signals. Through signal processing, the EMG signals are designed into three command signals to control shape deformations (such as flower-like structures) by three different hand gestures. Based on this working mechanism, an elastic metamaterial beam with tunable frequency responses is fabricated by bonding four PI/LIG/PDMS electrothermal actuators onto a host aluminum beam. The PI/LIG/PDMS electrothermal actuators are used as local resonators to control the elastic metamaterial beam's resonance frequencies. One end of the elastic metamaterial beam is fixed on a base. Then the base is excited by a shaker (sweep sine signal, 10–120 Hz). As shown in Figure 7C, in case I, all PI/LIG/PDMS electrothermal actuators are flat without actuation and induce a dip resonance around 111 Hz. In case III, all PI/LIG/PDMS electrothermal actuators are bent under actuation, and there is a largely attenuated peak at near 51 Hz. When it comes to case II, two pairs of PI/LIG/PDMS electrothermal actuators are flat, and the other two pairs of PI/LIG/PDMS electrothermal actuators are bent, leading to a bandgap behavior. This work shows tunable vibration and noise control ability, showing potential applications in suppressing environmental disturbances at ultralow frequency ranges. Besides, an LIG-based electrophysiological sensor is integrated on the PI surface of PI/LIG/PDMS electrothermal actuators (Figure 7D). The integration can be used to measure the electrocardiogram signals by warping the finger of a human.

#### Other smart devices

Graphene can be used as the electrode of dielectric elastomer actuators to prepare variable focus lens. Typically, Hwang et al. developed a variable focus lens using a few-layer-graphene (FLG) electrode.^61^ The top and bottom electrodes were fabricated by transferring FLG onto masked silicone substrates. Then, masks were removed, and the FLG/silicone/FLG actuator's transmittance was 57.0%. The FLG/silicone/FLG actuator is flat-shaped without actuation; the FLG/silicone/FLG actuator is curved-lens-shaped under actuation. Therefore, the focal length of the lens can be controlled by varying the input voltage between FLG/silicone/FLG actuators. This variable focus lens is promising applications in opto-electro-mechanical systems.

Besides, graphene can be used as the electrode of piezoelectric actuators to fabricate flexible loudspeakers. Bae et al. demonstrated a transparent piezoelectric actuator with G-P(VDF-TrFE)-G sandwiched structures.^100^ Due to the P(VDF-TrFE) fluoropolymer induced p-type doping effect, the sheet resistance of graphene decreases to 188 Ω/sq, which is a benefit for lower power consumption. The actuation mechanism is attributed to the inverse piezoelectric effect of P(VDF-TrFE). As a result, sounds or mechanical vibrations were produced by connecting the device to electronic circuitry. The actuator shows a broad frequency response within 1 to 3,000 Hz. Similarly, loudspeakers with G-PVDF-G structures have been successfully developed.^145^ These graphene-based actuators with high transparency and flexibility show great potential in easily integrating with transparent flexible systems.

**Figure 8:**
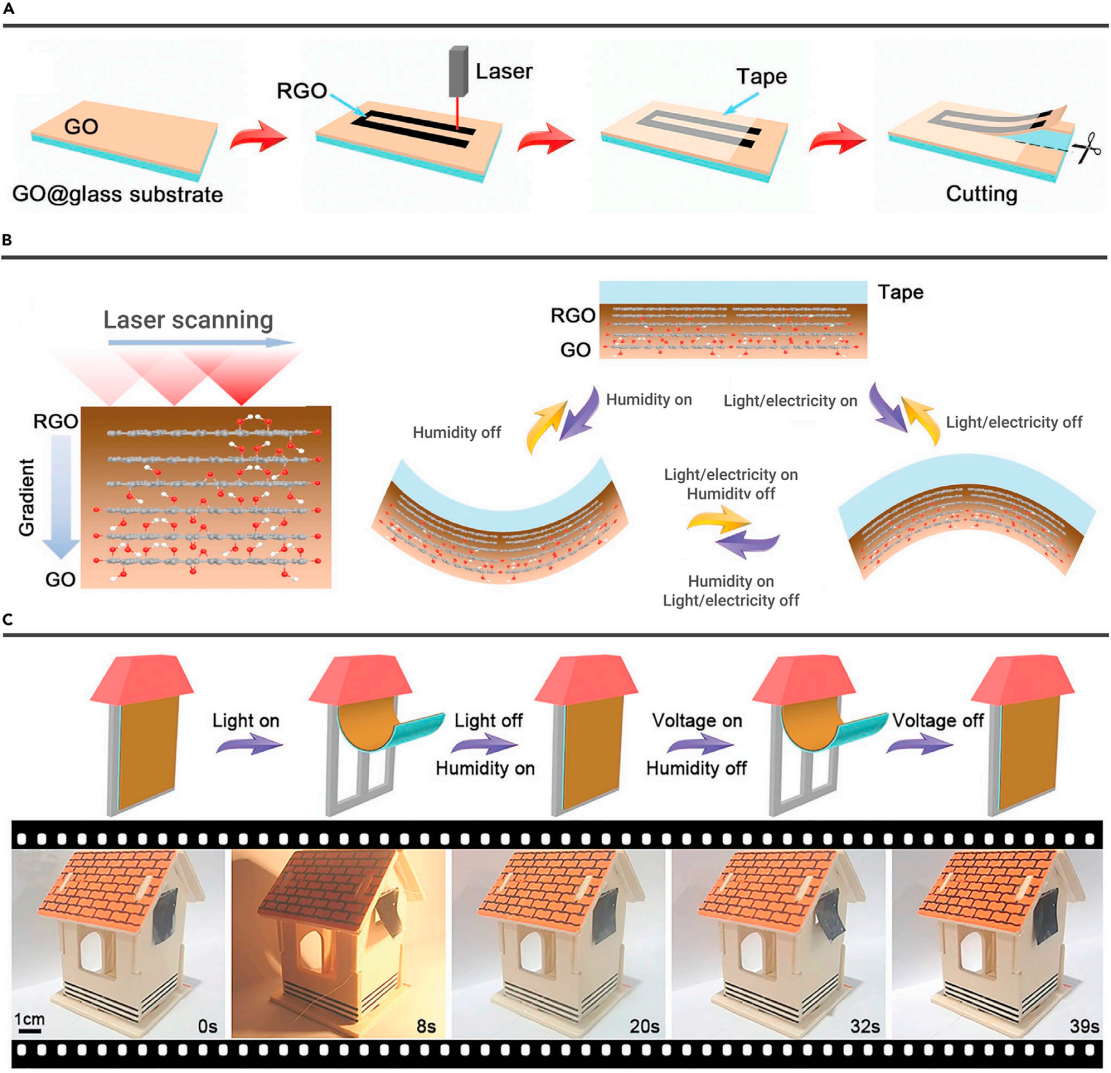
Smart devices (A) The preparation RGO/GO Janus Paper. (B) The schematic diagram of multiresponsive actuation. (C) Smart curtain model. Reproduced with permission from Ma et al.^126^ Copyright 2019, WILEY-VCH.

ERA integration with other external stimuli, such as light and moisture, can achieve cutting-edge applications. Ma et al. developed a tape/RGO/GO actuator-based smart curtain by the binary cooperative complementarity (Figure 8A).^126^ As shown in Figure 8B, the tape/RGO/GO actuators bend to the GO side under light and electricity stimuli because of a large CTE mismatch between the tape layer (≈137 ppm K^−1^) and the GO layer (0.85 ppm K^−1^).^146^ Tape/RGO/GO actuators bend to the tape side under moisture stimuli because the tape is inert to moisture, and the GO layer expands by absorbing water molecules. The tape/RGO/GO actuator was fixed one end on a house model's window. As a result, a smart curtain was demonstrated, which can sense weather changes (Figure 8C). The curtain can roll up under sunny weather by the light actuation. Then, the curtain can fall under rainy weather by the moisture actuation. At the same time, the closed curtain can open again under electricity actuation. Therefore, the smart curtain has abilities in opening and closing along with weather changes.

### Challenges and perspectives

In recent years, graphene and relative innovations have developed broad and important applications. Especially, graphene has become an attractive candidate for fabricating stimuli-responsive actuators. In this review, we summarize recent innovations of ERAs based on graphene, in which typical actuation mechanisms, including electrostatic actuation, electrothermal actuation, and ionic actuation, have been reviewed. In developing these ERAs, graphene and its derivatives prepared in different ways play an essential role. As compared with other carbon materials, the outstanding physical/chemical properties, the large specific surface, excellent stability, mechanical strength, and high electrical/thermal conductivity make graphene a promising candidate electrode for ERAs. In most cases, the use of graphene can promote the actuating performance significantly and facilitate the fabrication of ERAs. To make a comprehensive comparison of the performance of different types of graphene-based ERAs, we have summarized the reported critical properties of graphene ERAs in Table 1.

**Table 1:** Typical examples of ERAs based on graphene

Mechanisms	Structure	Fabrication method	Performances	Applications	Ref.
Electrostatic actuation	Mechanical exfoliation graphene	FIB etching	0.83 μm @ 100V	Nano gripper	Fujiwara et al.^102^
Electrostatic actuation	CVD-graphene/OCA/CVD-graphene	Roll to roll	∼1050 μm @ 3 kV	–	Kim et al.^99^
Electrostatic actuation	Epoxy-graphene-epoxy	Vacuum filtration	∼9.3 mm@ 12.5 kV	–	Yu et al.^147^
Electrostatic actuation	Charged GO	Solvent evaporation	40.1° @ 2.5 kV/cm	Smart radar, dancer	He et al.^104^
Electrostatic actuation	Carbon electrode/RGO@PDMS-PDMS/carbon electrode	Spin-coating	1.12 mm @ 63.4 MV/m	–	Zhang et al.^101^
Electrostatic actuation	RGO spring	Wrapping and annealing	210% @ 3.5 kV	Spring	Cheng et al.^103^
Electrothermal actuation	CVD-graphene/epoxy	Photolithography	4.5 μm @ 1.2 mW	Dragonfly wing	Zhu et al.^115^
Electrothermal actuation	RGO/PDA	Coating, UV radiation	0.37 cm^−1^ @ 0.74 A/mm^2^	Crawling robot	Liang et al.^107^
Electrothermal actuation	RGO/PE	Laser scribing	3 cm^−1^ @ 60V	Flytrap robot	Zhu et al.^124^
Electrothermal actuation	RGO/GO	Vacuum filtration	∼7.2 mm @ 20V	Artificial cilia	Bi et al.^116^
Electrothermal actuation	RGO/PDMS	Direct ink writing	300° @ 13.5V	Biomimetic hand	Li et al.^118^
Electrothermal actuation	RGO/PVDF	Drop-coating	14 mm @ 13V	Swimming robot	Xiao et al.^110^
Electrothermal actuation	RGO-AgNP/PI	Laser scribing	192° @ 28 V	Gripper	Wang et al.^123^
Electrothermal actuation	BOPP/RGO/GO	Direct laser writing	92° @ 60V	Smart curtain	Ma et al.^126^
Electrothermal actuation	PVDF/LIG/PI	Direct laser writing	2.5 cm^−1^ @ 14V	Gripper	Deng et al.^125^
Electrothermal actuation	PDMS/LIG/PI	Direct laser writing	3.3 cm^−1^ @ 30V	Origami, frog tongue, human-machine interaction	Ling et al.^39^
Ionic actuation	Au/GO-PSC-IL/Au	Casting	13.16 m^−1^ @ (10 V, 0.1 Hz)	–	Jeon et al.^148^
Ionic actuation	RGO/Nafion-BMIBF_4_/RGO	Laser scribing	4.26 mm @ (5 V, 0.01 Hz)	–	Kim et al.^127^
Ionic actuation	GM-NRGO/Nafion-EMIMBF_4_/GM-NRGO	Drop-coating	4 mm @ (3 V, 0.1 Hz)	Gripper	Tabassian et al.^141^
Ionic actuation	RGO-MWCNT/PVDF-BMIMBF_4_/RGO-MWCNT	Heat pressing	∼1.8 mm @ (2 V, 0.1 Hz)	–	Lu et al.^133^
Ionic actuation	RGO-AgNP/(PVDF-HFP)-BMIMBF_4_/RGO-AgNP	Heat pressing	2.6 mm @ (1 V, 8.33 Hz)	–	Lu et al.^131^
Ionic actuation	Th-SNG-PEDOT:PSS/SPBI-EMIMBF_4_/Th-SNG-PEDOT:PSS	Self-assembly	4.5 mm @ (1 V, 0.1 Hz)	–	Kotal et al.^129^
Ionic actuation	g-C_3_N_4_-NRGO-PEDOT:PSS/Nafion-EMIMBF_4_/g-C_3_N_4_-NRGO-PEDOT:PSS	Drop-coating	6.5 mm @ (0.5 V, 0.1 Hz)	Fish fin	Kotal et al.^140^
Ionic actuation	pMoS_2_-nSNrGO-PEDOT:PSS/Nafion-EMIMBF_4_/pMoS_2_-nSNrGO-PEDOT:PSS	Casting	9.89 mm @ (0.5 V, 0.1 Hz)	Robotic fingers	Manzoor et al.^139^
Ionic actuation	PGNR-G-PEDOT:PSS/Nafion-EMIMBF_4_/PGNR-G-PEDOT:PSS	Coating	17.4 mm @ (0.5 V, 0.1 Hz)	Flytrap robot	Kotal et al.^130^

For ERAs based on electrostatic actuation, the working mechanism is the interaction of electric charge-induced attractions or repulsions between graphene electrodes. Here, graphene can either serve as the electrode material or work as a dopant to increase the dielectric constant of the dielectric elastomer, which improves the performance of dielectric elastomer actuators. Generally, dielectric elastomer actuators show high-frequency response, but suffer from high-applying voltages and relatively small displacement, which more or less limits their applications. Nevertheless, due to the high response rate, electrostatic actuators have been successfully employed for artificial muscles (e.g., nano-gripper) and human-machine interaction (e.g., radar, dancers). Nevertheless, for much broader applications, their performance still needs further improvements to achieve much lower driving voltages and larger displacements. Recently, spring-shaped micro-electrostatic actuators that can work under lower voltage (110V) have been reported, which show much larger displacements (elongate rates, ∼210% per s). These works provide a hint that electrostatic actuators can be optimized by designing novel device structures, reducing the device dimensions, and using high-quality graphene as electrodes.

In the case of graphene-based electrothermal actuators, the device structure is a common bilayer or multilayer, in which graphene electrodes convert electrical energy into heat, inducing the thermal expansion of coupled material layers. In addition to the electrodes, graphene plays very important roles, including electrothermal generation, heat conduction, and negligible thermal expansion. Generally, electrothermal actuators feature a very simple device structure and preparation process. Many technologies, including drop-coating, ink printing, vacuum filtration, photopolymerization, and casting, can be employed to prepare graphene electrothermal actuators. Nevertheless, this actuation scheme usually suffers from low energy conversion efficiency, and the actuation voltage is still more than 10 volts. To reduce the actuation voltage and increase displacements, improvements can be made by using graphene electrodes with more obvious electrothermal effects and active materials with larger CTEs. Besides benefiting from the patterned and periodically structured graphene electrodes (e.g., LRGO or LIG), electrothermal actuators also enable complex shape morphing beyond simple bending. To get better control over the electrothermal deformation, graphene electrodes can be combined with shape-memory materials. In that case, the temperature-determined reversible shape transition between different states may lead to broad applications.

Ionic actuators generally consist of metal electrodes and electrolytes. However, in most cases, metal electrodes suffer from poor stability considering the leakage of electrolytes and mobile ions. Recently, graphene has emerged as an appealing alternative due to its chemical stability, superhydrophobicity, high electrical conductivity, specific surface area, and flexibility. Generally, ionic actuators can be prepared using different graphene electrodes. RGO-based electrodes can be readily prepared, while they suffer from relatively low conductivity. To make a further improvement, strategies including micro/nano-structuring, heteroatom doping, or nanoparticle/wire hybriding and the construction of P-N junction have been developed to enhance the electrochemical properties. Besides, CVD-grown graphene has been also used as the electrodes to increase the electrical conductivity. Consequently, ionic actuators with fast response (<1 s), excellent stability, and low voltage actuation (<1 V) have been developed, revealing the great potential for artificial fingers, fish fin, and insect wings.

The use of graphene for developing ERAs shows distinct advantages and several shortcomings. In most cases, graphene works as an electrode. As compared with other carbon electrodes, for instance CNTs, the merits of graphene lie in the unique 2D nanosheet structure. It can form very thin electrodes with high transparency, flexibility, mechanical strength, and tunable chemical/physical properties. On the contrary, the shortcomings of graphene are also obvious, for example, the use of graphene may increase the cost of devices because all kinds of graphene electrodes, including RGO, LIG, and CVD-graphene, cannot be produced by industry at present. Besides, graphene electrodes prepared different ways may have distinct properties. For instance, RGO electrodes usually have relatively lower conductivity than CVD-graphene, but more electrochemically active sites. The patterning/structuring of RGO and LIG is much easier than CVD-graphene, since the former type of graphene is generally multilayer graphene, whereas the later one is single- or few-layer graphene. In this regard, it is not accurate to simply discuss the shortcomings of the graphene without pointing out which types of graphene. Different graphene electrodes may be suitable for different ERAs. Currently, an obvious limitation for practical use of graphene might be the uniformity of graphene electrodes prepared through different methods and even through the same method but from different batches. For example, in the case of RGO electrodes, the size of raw graphite, the oxidation condition, and the reduction methods are all crucial factors for the quality of RGO samples. Consequently, it is quite urgent to establish relevant standards for both graphene preparation and the quality of the resultant products. To make a comprehensive comparison of the performance of different types of electrode materials for ERAs, we have summarized typical examples of ERAs based on graphene and other conductive materials in Table 2.

**Table 2:** Typical examples of ERAs based on graphene and other conductive materials

Mechanisms	Electrode materials	Size	Deformation	Response time	Conductivity	Ref.
Electrothermal actuation	CVD-graphene	200 μm × 180 μm	4.5 μm @ 1.2 mW	0.02 s (1V, DC)	∼200 Ω/sq	Zhu et al.^115^
Electrothermal actuation	RGO-AgNP	∼26 mm × 12 mm	192° @ 28 V	6 s (28V, DC)	121.3 Ω/sq	Wang et al.^123^
Electrothermal actuation	LIG	∼50 mm × 5 mm	3.3 cm-1 @ 30V	3 s (12V, DC)	∼5000 S/m	Ling et al.^39^
Electrothermal actuation	MXene	-	122° @ 6V	40 s (4V, DC)	186000 S/m	Sang et al.^149^
Electrothermal actuation	CNT	35 mm × 9 mm	180° @ 25V	12s (25V, DC)	30000 S/m	Li et al.^150^
Electrothermal actuation	AgNW	-	∼2.5 cm-1 @ 15V	∼30 s (15V, DC)	7.6 Ω/sq	Kim et al.^151^
Electrothermal actuation	AgNW/PEDOT:PSS/EG	45 mm × 9 mm	1.07 cm-1 @ 7V	<20 s (8V, DC)	∼2 Ω/sq	Amjadi et al.^152^
Ionic actuation	RGO	∼30 mm × 5 mm	4.26 mm @ (5 V, 0.01 Hz)	40 s (6V, DC)	31500 S/m	Kim et al.^127^
Ionic actuation	NS codoped graphene/	-	8.2 mm @ (0.5 V, 0.1 Hz)	5 s (0.5V, DC)	-	Nguyen et al.^153^
	PEDOT:PSS					
Ionic actuation	PGNR-G-PEDOT:PSS	24 mm × 4 mm	17.4 mm @ (0.5 V, 0.1 Hz)	700 ms (0.5V, DC)	956 S/cm	Kotal et al.^130^
Ionic actuation	MXene	40 mm × 5 mm	0.038 mm-1 @ (1.2V, 0.001 Hz)	-	∼4000 S/cm	Pang et al.^154^
Ionic actuation	MXene/PEDOT:PSS	10 mm × 3 mm	-	∼1 s (0.5V, DC)	14590.56 S/cm	Umrao et al.^155^
Ionic actuation	CNT	4 mm × 1 mm	∼800 μm @ (3V, 0.1Hz)	∼5 s (3V, AC)	-	Fukuda et al.^156^
Ionic actuation	SWCNT/W18O49NW	20 mm × 3 mm	1.83 mm @ (1.8V)	1.4 s (1.8V)	1590 S/cm	Li et al.^157^

At present, despite the rapid advancements, graphene-based ERAs are still at an early stage. Current challenges for developing high-performance ERAs mainly lie in the following three aspects: (1) high-quality graphene electrodes with high uniformity that can be practically applied to ERAs are still rare; (2) the optimization of device structures and performance; (3) the integration of ERAs with control circuits. Besides, graphene-based ERAs may feature ultrathin device structure, light weight, flexibility, and mechanical robustness, as compared with ERAs based on other materials. Therefore, another challenging task for ERAs is their broad applications in different fields. Actually, the issues mentioned above are not independent but inter-constraint with each other. Consequently, the development of graphene-based ERAs depends on the overall progress of material sciences, nanotechnologies, and electronics. It is possible that with the improvement of graphene preparation/processing technologies and the innovation of advanced flexible electronics, graphene-based ERAs may develop rapidly and find broad applications in the near future.

## Lead contact website

http://cufo.jlu.edu.cn/index.htm.
